# LIVING ALONE WITH DEMENTIA AND NAVIGATING ESSENTIAL TRANSPORTATION NEEDS

**DOI:** 10.1093/geroni/igad104.3766

**Published:** 2023-12-21

**Authors:** Mary Nemec, Laura Girling

**Affiliations:** Towson University, Elkridge, Maryland, United States; Towson, Towson, Maryland, United States

## Abstract

Transportation serves a basic human need for older adults and is firmly linked to independence, quality of life, and access to essential services (e.g., medical, nutritional, social). The matter of transportation and driving reduction/cessation is especially challenging in regard to people with Alzheimer’s and related dementias (ADRD). Many individuals with ADRD rely on cohabitating caregiver(s) (e.g., spouse, partner, adult children) to arrange and execute essential transportation needs. However, little is known about how individuals with ADRD who reside alone in the community navigate required transportation. In order to address this gap in the literature, this paper combines narrative data from two ongoing National Institutes of Health (NIH)-funded interview-based protocols that focus on understanding the needs of community-dwelling older adults with ADRD and a collateral informant (N=84). Interview data underwent thematic analyses. Findings indicate four overarching themes: 1. Nuances of ridesharing apps, 2. Family/fictive kin transportation network(s), 3. Unpredictable public transportation, 4. Unmet needs exacerbated by inadequate transportation, and 5. Balancing driving autonomy and risk of harm to self/others. These thematic illations have implications to assist development of strategies for continued mobility and appropriate access to required services for those who reside alone with ADRD, despite changing needs and capabilities. Historically, the focus on transportation among those with ADRD has centered on those with a cohabitating caregiver, however, our findings provide insights into the nuances of how this understudied subpopulation with ADRD experience and navigate their mobility needs.

